# Hippocampal Over-Expression of Cyclooxygenase-2 (COX-2) Is Associated with Susceptibility to Stress-Induced Anhedonia in Mice

**DOI:** 10.3390/ijms23042061

**Published:** 2022-02-13

**Authors:** Tatyana Strekalova, Dmitrii Pavlov, Alexander Trofimov, Daniel C. Anthony, Andrei Svistunov, Andrey Proshin, Aleksei Umriukhin, Alexei Lyundup, Klaus-Peter Lesch, Raymond Cespuglio

**Affiliations:** 1 Department of Psychiatry and Neuropsychology, School for Mental Health and Neuroscience, Maastricht University, 6229 ER Maastricht, The Netherlands; alexander.n.trofimov@gmail.com (A.T.); kplesch@mail.uni-wuerzburg.de (K.-P.L.); 2 Laboratory of Psychiatric Neurobiology, Institute of Molecular Medicine and Department of Normal Physiology, Sechenov First Moscow State Medical University, 119991 Moscow, Russia; dmitrii.pavlov1@ucalgary.ca (D.P.); daniel.anthony@pharm.ox.ac.uk (D.C.A.); svistunov@sechenov.ru (A.S.); alum1@yandex.ru (A.U.); cespuglio@univ-lyon1.fr (R.C.); 3 Hotchkiss Brain Institute, Alberta Children’s Hospital Research Institute, University of Calgary, Calgary, AB T2N 4N1, Canada; 4 P.K. Anokhin Research Institute of Normal Physiology, 125315 Moscow, Russia; proshin_at@mail.ru; 5 Research and Educational Resource Center for Cellular Technologies, Peoples’ Friendship University of Russia, 117198 Moscow, Russia; lyundup@gmail.com; 6 Division of Molecular Psychiatry, Center of Mental Health, University of Würzburg, 97080 Wuerzburg, Germany; 7 Centre de Recherche en Neurosciences de Lyon (CRNL), 69500 Bron, France

**Keywords:** major depression, inducible cyclooxygenase-2 (COX-2), hippocampus, anhedonia, chronic stress, stress resilience, fear conditioning, celecoxib, citalopram, mouse

## Abstract

The phenomenon of individual variability in susceptibility/resilience to stress and depression, in which the hippocampus plays a pivotal role, is attracting increasing attention. We investigated the potential role of hippocampal cyclooxygenase-2 (COX-2), which regulates plasticity, neuroimmune function, and stress responses that are all linked to this risk dichotomy. We used a four-week-long chronic mild stress (CMS) paradigm, in which mice could be stratified according to their susceptibility/resilience to anhedonia, a key feature of depression, to investigate hippocampal expression of COX-2, a marker of microglial activation Iba-1, and the proliferation marker Ki67. Rat exposure, social defeat, restraints, and tail suspension were used as stressors. We compared the effects of treatment with either the selective COX-2 inhibitor celecoxib (30 mg/kg/day) or citalopram (15 mg/kg/day). For the celecoxib and vehicle-treated mice, the Porsolt test was used. Anhedonic (susceptible) but not non-anhedonic (resilient) animals exhibited elevated COX-2 mRNA levels, increased numbers of COX-2 and Iba-1-positive cells in the dentate gyrus and the CA1 area, and decreased numbers of Ki67-positive cells in the subgranular zone of the hippocampus. Drug treatment decreased the percentage of anhedonic mice, normalized swimming activity, reduced behavioral despair, and improved conditioned fear memory. Hippocampal over-expression of COX-2 is associated with susceptibility to stress-induced anhedonia, and its pharmacological inhibition with celecoxib has antidepressant effects that are similar in size to those of citalopram.

## 1. Introduction

Major depressive disorder (MDD) is a common mental illness that markedly diminishes quality of life and has a profound medical and socioeconomic burden [1,2,3]. While MDD was identified by the World Health Organization (WHO) as a “global crisis” a decade ago [4], the COVID-19 outbreak has aggravated the situation [5,6]. The ongoing pandemic has been projected to have impact on the incidence of MDD that affects not only the patient, but also their relatives, caregivers, and the wider community [7]. Despite the variety of therapeutic regimens available for depression, many of them appear to be effective in half of patients or less [8,9,10,11] and cause significant side effects [12,13,14]. At the same time, the development of new, effective antidepressant treatment strategies is an ongoing need in neuropsychopharmacology [15,16].

Currently, the predominant treatment for MDD remains monotherapy with classic antidepressants—i.e., targeting monoaminergic neurotransmission in the brain [14,17]. Many treatment options based on other mechanisms have been proposed, with targets ranging from neurotrophin- and immune-related molecules, to neurodevelopmental, glutamatergic, GABAergic, metabolic mechanisms, and more recently, gut microbiota [18]. The need for a new mechanistic framework for treating MDD is urgently required, but it has been challenging, as translation from clinically relevant animal models to clinical application has been problematic [19,20,21,22]. Among the novel compounds with antidepressant activities, the use of anti-inflammatory drugs, targeting low-level inflammation, a well-established feature of MDD [23,24,25,26,27], might be of particular value owing to the long-established clinical experience with these widely prescribed drugs [28].

Increased cyclo-oxygenase (COX) activity is a well-established feature of neuroinflammation, and the inducible isoform COX-2 in particular seems to play the predominant role in the CNS [29,30,31]. COX-2 is also constitutively expressed throughout the forebrain in discrete populations of neurons and is particularly enriched in the hippocampus and cortex [32], where it appears to contribute to fundamental brain functions, such as synaptic activity and memory consolidation [33,34]. Under resting conditions, however, it is not expressed by glial or endothelial cells [35,36].

COX converts arachidonic acid to prostaglandin (PG) G2, which, in turn, is converted to PGH2 and then to prostaglandins, prostacyclins, and thromboxanes, among which PGE2 regulates many physiological and pathological functions [30,37]. COX-2 expression is regulated by synaptic plasticity and depends on glucocorticoids, and is, therefore, regarded as important for dendritic remodeling as part of the stress response and associated with neuropsychiatric disorders [32,36,38]. In the hippocampus, COX-2 basal expression is positively regulated by NMDA receptor-dependent synaptic plasticity and is restricted to the CA3 area, but under stressful conditions or global ischemia, COX-2 can also be upregulated in the CA1 area and the dentate gyrus, causing neuronal death in those regions, which is prevented by the administration of either glucocorticoids or COX-2 selective inhibitors [32,39,40]. Under pathological conditions, the over-expression of COX-2 results in increased synthesis of prostaglandins, including PGE2 [38], which, in turn, increases the sensitivity of tissues to catecholamines, stimulates the activity of the HPA axis via corticotropin-releasing factor [41,42,43], and leads to a surge in the production of pro-inflammatory cytokines, e.g., interleukin (IL)-1ß, IL-6, and tumor necrosis factor (TNF) [44,45]. The latter changes can elevate the activity of the serotonin transporter SERT [46], increase the affinity of the serotonin receptor 5-HT1A [47], and alter tryptophan metabolism via indoleamin-2,3-dioxigenase (IDO) mechanism [48] resulting in depressive-like “sickness behavior” [49,50].

COX-2 upregulation was shown to be implicated in several neuropsychiatric diseases, including MDD, schizophrenia, brain ischemia, and neurodegenerative disorders [51,52,53]. A number of findings suggest a role of altered COX-2-mediated molecular cascades in MDD. Increased expression and turnover of COX-2 protein, COX-2 activity, and elevated PGE2 were found to be associated with MDD symptoms, whereas COX-1 protein remained unaltered [51,54]. Earlier studies suggested that the stimulation of prostaglandin synthesis by prolactin or other hormones can contribute to mood disorders [55]. PGE2 is reported to be increased in the plasma and cerebrospinal fluid of depressed patients [56,57].

Furthermore, pre-clinical genetic and pharmacological studies have implicated PG- PGE2, PGD2, PGF2a, PGI2, and thromboxane-A2, all synthesized downstream of COX-2, in the mechanisms of the depressive syndrome [58,59,60,61]. Brain over-expression of PGE2 has been associated with depressive-like behavior in a chronic mild stress (CMS) model [59,61], in a model of systemic inflammation [61], following a forced swim (Porsolt) test [62], and in the rat bulbectomy model of depression [60,63]. Roles for COX-2-mediated brain increases of PGE2, dendritic dysfunction, and neuronal injury were reported in the rat bulbectomy model [61].

Recent meta-analyses of clinical studies in depressed patients, including retrospective cohort studies (RHSs), randomized controlled trials (RCTs), and nested case-control studies (NCCSs) have demonstrated the therapeutic efficacy of pharmacological inhibition of COX-2 with its selective inhibitor celecoxib. Combined treatment with celecoxib increases the effectiveness of established antidepressant compounds in patients with various forms of depression when used as an augmentation strategy together with reboxetine, fluoxetine, and other antidepressants [50,64,65,66,67]. Treatment with celecoxib was specifically shown to normalize dysregulated cortisol secretion in MDD patients [68,69].

While the meta-analysis on the use of COX-2 inhibitors in MDD found an overall benefit of celecoxib add-on therapy, some studies have failed to support these findings [70,71]. Similarly, pre-clinical studies have generated controversies on the effects of COX-2 inhibition. For example, suppression of COX-2 in rodent studies resulted in increased Th1 immune responses and glial cell activation [72,73,74]. Mice genetically deficient for COX-2 revealed increased rates of neuronal damage, microglia, and astrocyte activation; over-production of markers of inflammation; abnormal oxidative and nitrosative stress; and an abnormal response to celecoxib [72,73].

The controversial effects of COX-2 inhibition are attributed to the complex roles of this enzyme in normal brain functions and the stress response, and the broad spectrum of COX-2 activities [38]. While the function of COX-2 has been investigated in rodent depression models, its role in individual susceptibility to MDD-like syndrome precipitated by stress has not been not addressed. Concurrently, the phenomenon of individual differences in susceptibility versus resilience to stress and depression is attracting increasing attention [75,76,77]. Several important molecular and cellular mechanisms constituting the biological basis of these phenomena have been described [78,79], and the hippocampus has been argued to be the structure within the brain that plays the most important role in governing an individual’s susceptibility or resilience to stress-induced depression and mental disorders in general [80,81,82,83].

To address the potential role of hippocampal COX-2 in the mechanisms of the susceptibility to MDD-like behavior, we used a variant of the original CMS model [84,85] that is based on the induction of decreased sensitivity to reward (anhedonia) as the core depressive symptom [86,87], and on the previously observed individual susceptibility of 50–70% of C57BL6 mice to this condition [88,89,90,91]. In this model, the anhedonic (susceptible) state in stressed mice is defined by a decrease in sucrose preference that is not exhibited by non-stressed control animals; typically, it is not displayed by all stressed mice. As such, the non-anhedonic (resilient) mice can be regarded as an internal control for the effects of stress that are not related to depressive-like changes [22,88,92].

**Figure 1:**
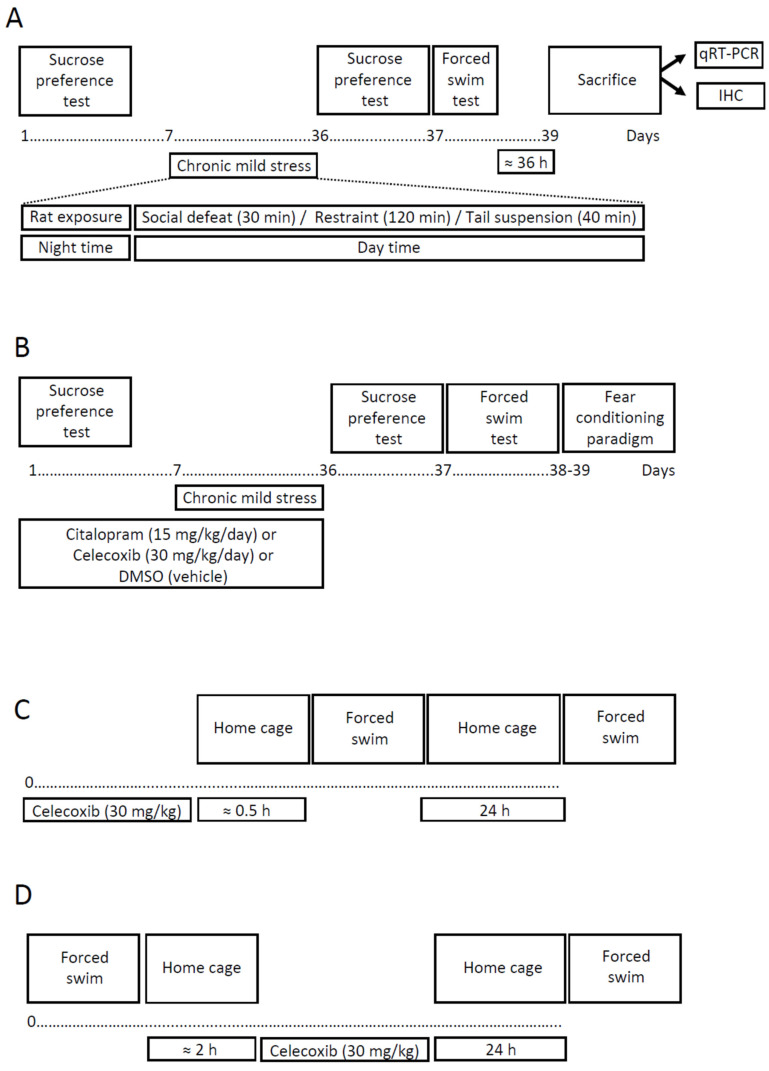
**Experiment design**. Chronic mild stress without pharmacological interventions (**A**) or with citalopram or celecoxib (**B**) involved 4 weeks with semi-random alternations of stressors. Forced swimming studies with celecoxib treatment 0.5 h before (**C**) or 2 h after (**D**) the first swimming session were carried out within 24 h. In experiments A and B, groups were balanced by mouse preference for sucrose before the chronic mild stress procedure. Following the post-stress behavioral test battery, mouse brains from experiment A were used for qRT-PCR and immunohistochemical studies. qRT-PCR—quantitative reverse transcription polymerase chain reaction; IHC- immunohistochemistry; see aslo the ms text.

C57BL6 mice underwent rat exposure, restraints, tail suspension, and social defeat for four weeks and were assigned to the susceptible or resilient to anhedonia groups as described elsewhere [88,93,94]. They were studied for floating behavior and hippocampal expression of COX-2, using PCR and immunohistochemical methods (Figure 1A). Additionally, Iba-1, as a marker of microglial cells, and Ki67, as a marker of cell proliferation, along with the markers for neurons and for cell nuclei were investigated, as pro-inflammatory changes are known to accompany suppressed neurogenesis under conditions of stress [95,96]. In a downstream CMS study, mice received celecoxib (30 mg/kg/day), or citalopram, an antidepressant of SSRI class (15 mg/kg/day), or DMSO-vehicle via i.p. injections for one week prior to the onset of the stress and then for the entire stress period, or they were not treated (Fig. 1 B; [93,97]). To assess hippocampus-dependent functions, contextual fear conditioning memory was investigated [96,98]. Finally, a group of mice received a single i.p. injection of celecoxib (30 mg/kg/day) prior to (Figure 1 C) or following (Figure 1 D) swim session in the Porsolt paradigm [99,100]. 

## 2. Results

### 2.1. Expression of COX-2 in the Hippocampi of CMS Mice

**Figure 2:**
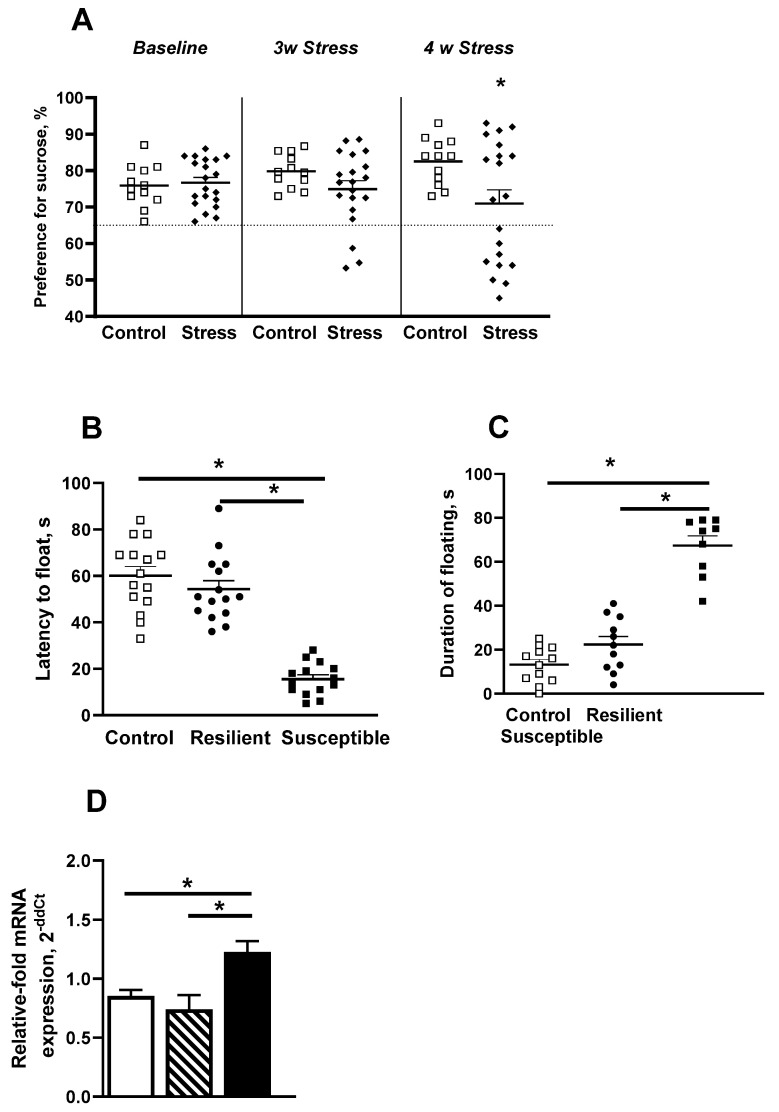
Behavioral and biochemical stratification of chronically stressed animals. (**A**) Preference for sucrose, measured one week before the start of chronic mild stress and 3 and 4 weeks later. A 65% preference was set as a criterion of anhedonia. Fourth week of stress is an optimum adversity duration to stratify animals into two distinct phenotypes, susceptible and resilient to anhedonia (* *p* < 0.05 vs. control, two-way ANOVA and post hoc Tukey test). (**B**) Susceptible-to-anhedonia animals had decreased latency before floating and (**C**) increased duration of floating (* *p* < 0.05 vs. control and resilient mice, one-way ANOVA and post hoc Tukey test). (**D**) COX-2 mRNA expression in hippocampus was upregulated in susceptible animals (* *p* < 0.05 vs. control, one-way ANOVA and post hoc Tukey test). Bars are mean ± SEM. ‘Open square’ symbols stand for non-stressed group, ‘diamond’ symbols indicate stressed mice, ‘circle’ symbols indicate stressed resilient animals, ‘filled squares’ stand for anhedonic stressed group.

In the CMS study, 20 mice were assigned to the chronic stress procedure and 12 animals formed a non-stressed control group. The 4-week stress procedure caused a significant reduction in sucrose preference in the stressed mouse group, as shown by two-way ANOVA (F_1,90_ = 6.029; *p* = 0.016) and Tukey post hoc test (*p* = 0.028; Figure 2A). According to the 65% criterion for sucrose preference that was applied [88,92], nine out of 20 stressed mice (45%) showed a sucrose preference below 65% and were defined as exhibiting anhedonia. The rest of the stressed animals, 11 out of 20 (55%), were considered to be non-anhedonic. Anhedonic mice displayed lower latency before floating (F_2,41_ = 51.66, *p* < 0.0001) and elevated duration of floating in the forced swim test as compared to control and non-anhedonic animals, as shown by one-way ANOVA (F_2,29_= 65.54, *p* < 0.0001) and Tukey test (*p* < 0.0001 for all the cases; Figure 2C). One-way ANOVA revealed a significant difference in the relative expression of COX-2 mRNA in the hippocampus of stressed mice (F_2,27_ = 6.9; *p* = 0.038; Figure 2D). Anhedonic mice exhibited overexpression of COX-2 in comparison with non-anhedonic stressed and control animals (*p* = 0.035 and *p* = 0.03, respectively, Tukey test).

### 2.2. Immunohistochemistry for COX-2, Iba-1, and Ki67 Expression in the Hippocampi of Mice Resilient and Susceptible to CMS-Induced Anhedonia

**Figure 3:**
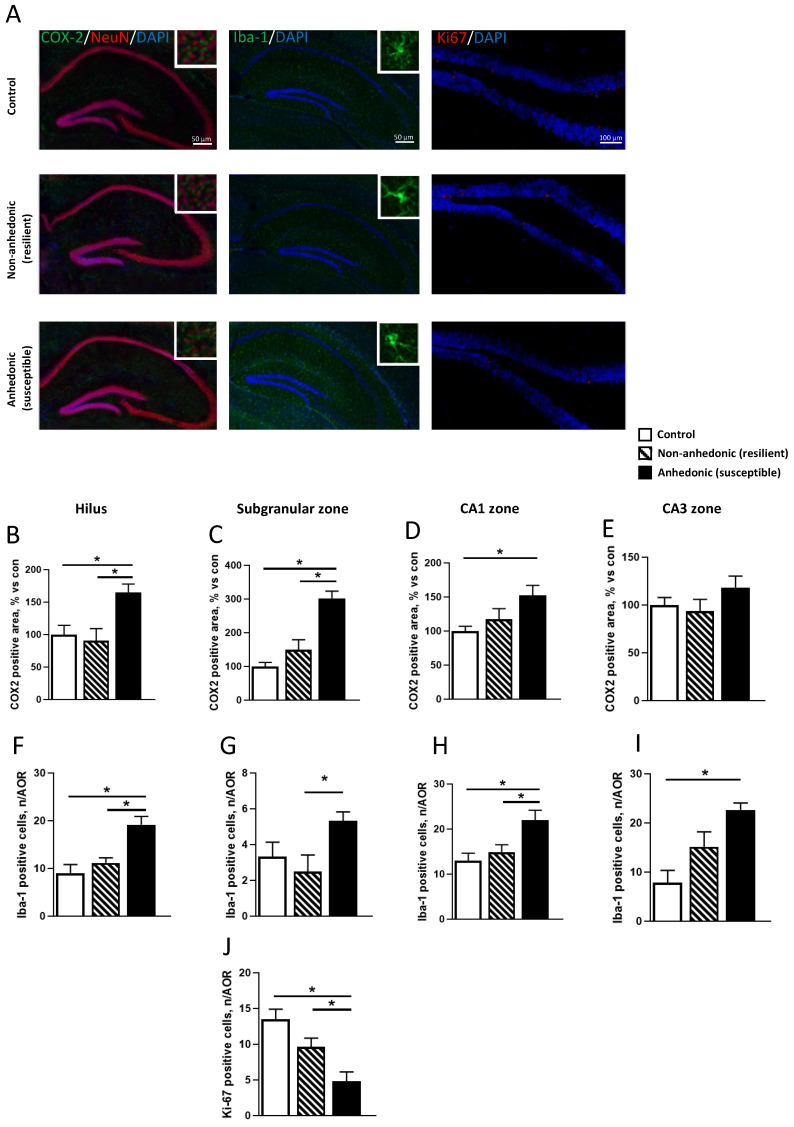
Immunohistochemical analysis of COX-2 and markers of microgliosis and neurogenesis in the hippocampal formation of susceptible and resilient mice. (**A**) Immunohistochemical staining of COX-2. NeuN, a neuronal marker; Iba-1, a marker of microglia; and Ki67, a marker of cellular proliferation. DNA-labeling dye DAPI was used to detect nuclei. Susceptible animals had greater COX-2 positive areas in the (**B**) hilus, (**C**) subgranular zone, and (**D**) CA1 region, but not in the (**E**) CA3 zone. Iba-1 positive cells were outnumbered in the (**F**) hilus, (**G**) subgranular zone, (**H**) CA1 zone, and (**I**) CA3 zone of hippocampus of susceptible animals. (**J**) Chronic stress diminished the number of Ki67 positive cells in the subgranular zone in susceptible animals. * *p* < 0.05 vs. control and resilient mice, one-way ANOVA and post hoc Tukey test. Bars are mean ± SEM.

Mice that underwent CMS exhibited a significant group difference in hippocampal COX-2 content according to one-way ANOVA in the hilus area (F_2,15_ = 6.89, *p* = 0.075, Figure 3B) and subgranular zone (F_2,15_ = 22.91, *p* < 0.0001, Figure 3C). COX-2 upregulation in both hippocampal areas was observed in the anhedonic group as compared to the non-anhedonic and control animals (*p* = 0.0104 and *p* = 0.023 in hilus, *p* = 0.0005 and *p* < 0.0001 in the subgranular zone, Tukey post hoc test). For the CA1 hippocampal zone, one-way ANOVA revealed significant group differences (F_2,15_ = 4.23, *p* < 0.0001, Figure 3D), but for the CA3 area no differences were observed (F_2,15_ = 1.38, *p* = 0.281, Figure 3E). Subsequently we examined population of Iba-1-positive microglial cells in the same areas, and one-way ANOVA revealed group differences for hilus area (F_2,15_= 11.34, *p* = 0.001, Figure 3F), subgranular zone (F_2,15_ = 3.65, *p* < 0.0508, Figure 3G), CA1 (F_2,15_ = 6.55, *p* < 0.009, Figure 3H), and CA3 area (F_2,15_ = 9.32, *p* < 0.0023, Figure 3I). In the hilus and CA1 zone, anhedonic mice had outnumbered microglia in comparison with non-anhedonic and control mice (*p* = 0.0076 and *p* = 0.0011 for hilus; *p* = 0.0039 and *p* = 0.0099 for CA1 area); and in the subgranular zone there was a significant increase in the microglial population as compared to non-anhedonic mice, but not compared to control animals (*p* = 0.046 and *p* = 0.185). In the CA3 zone, we observed a significant increase in microglial cells as compared to control mice, but not compared to non-anhedonic ones (*p* = 0.0017 and *p* = 0.106). To examine hippocampal neurogenesis in the subgranular zone, we used Ki67, whose expression significantly varied across CMS groups (F_2,15_ = 11.19, *p* = 0.0011, one-way ANOVA; Figure 3J). The lowest Ki67 content was observed in the anhedonic group (non-anhedonic and control animals, *p* = 0.0468 and *p* = 0.0008).

### 2.3. Effects of Chronic Treatment with Celecoxib and Citalopram on the Development of Stress-Induced Anhedonia and Depressive-like Syndrome

**Figure 4:**
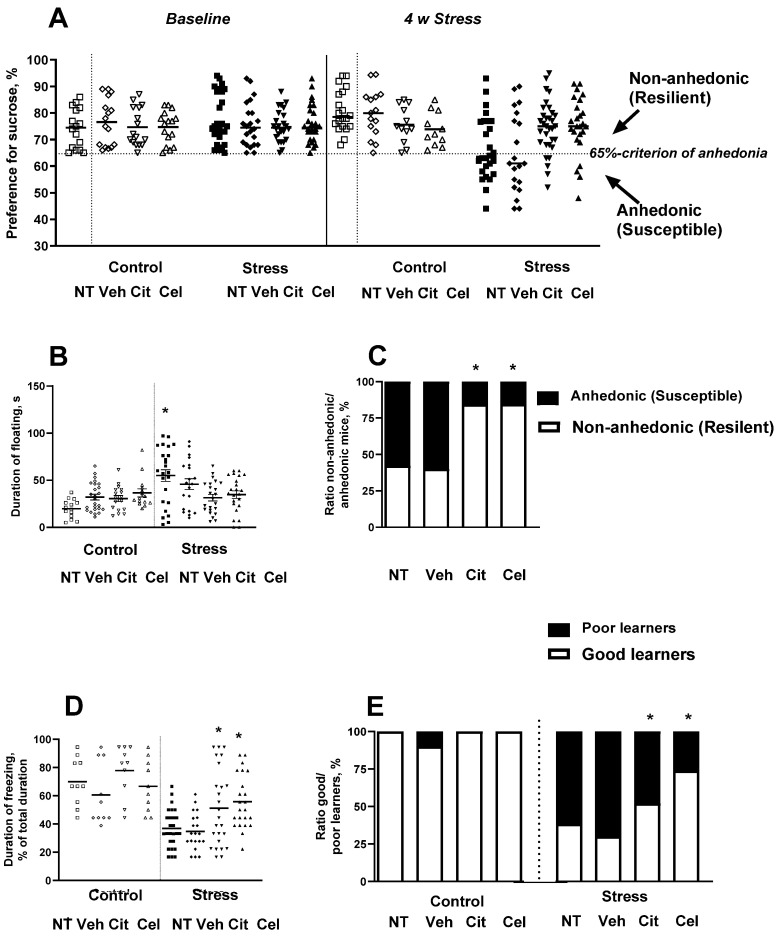
Effects of pharmacological intervention with citalopram or celecoxib on stratification of chronically stressed animals. (**A**) Preference for sucrose, measured one week before the start of chronic mild stress and 4 weeks thereafter, with and without citalopram and celecoxib. A 65% preference was set as a criterion of anhedonia. Both pharmacological agents did not affect sucrose preference in naïve or vehicle-treated non-stressed mice. The population of susceptible animals was decreased in both treated groups as compared with untreated and vehicle-treated stressed groups (* *p* < 0.05 vs. respective control, two-way ANOVA and post hoc Tukey test). Fourth week of stress is an optimum adversity duration to stratify animals into two distinct phenotypes, susceptible and resilient to anhedonia (* *p* < 0.05 vs. control, two-way ANOVA and post hoc Tukey test). (**B**) Citalopram- and celecoxib-treated groups of stressed animals did not demonstrate increased duration of floating as untreated and vehicle-treated did (* *p* < 0.05 vs. control and resilient mice, two-way ANOVA and post hoc Tukey test). (**C**) Ratio of non-anhedonic to anhedonic animals was reversed by both citalopram and celecoxib treatments (* *p* < 0.05 vs. respective control, two-tailed Fisher’s exact test). (**D**) In the fear conditioning paradigm, citalopram and celecoxib prevented a decrease in freezing duration observed in stressed not treated or vehicle treated mice (* *p* < 0.05 vs. control and resilient mice, two-way ANOVA and post hoc Tukey test). (**E**) Ratio of good and poor learners was reversed by both citalopram and celecoxib treatments (* *p* < 0.05 vs. respective control, two-tailed Fisher’s exact test). NT- no treatment, Veh- vehicle, Cit- citalopram, Cel- celecoxib.

After the termination of stress procedure, all stressed mice were classified as either non-anhedonic or anhedonic (*see below*). In the sucrose preference test, two-way ANOVA revealed a significant group difference (F_15,305_ = 8.729, *p* < 0.0001, Figure 4A). Post hoc analysis revealed a significant decrease in sucrose preference in the vehicle-treated group (*p* = 0.018, Tukey test) in comparison with untreated animals. The administration of citalopram or celecoxib prevented this decline (*p* = 0.028 and *p* = 0.032) as compared with the stressed untreated and stressed vehicle-treated animals, respectively. For the forced swim test, two-way ANOVA revealed significant group differences in the duration of floating (F_7,133_ = 15.228, *p* < 0.0001, Figure 4B). Post hoc analysis revealed a significant increase in floating duration in the untreated group (*p* = 0.002). Citalopram- and celecoxib-treated mice ameliorated the increased floating duration in this test (*p* = 0.518 and *p* = 0.455), as compared with the control untreated and control vehicle-treated animals, respectively. A two-tailed exact Fisher test showed that percentages of anhedonic mice in the citalopram-treated and celecoxib-treated stressed groups were significantly lower than those of vehicle-treated and untreated groups, respectively (*p* < 0.0001 and *p* < 0.0001, respectively). In the untreated group, 15 out of 26 mice were anhedonic (57.69%); in the citalopram-treated stressed group, 5 out of 32 mice were anhedonic (15.62%); in the celecoxib-treated mice, 4 out of 26 animals were anhedonic (15.38%); and in the vehicle-treated stress group, 12 out of 20 mice were anhedonic (60%; Figure 4C). 

In the fear conditioning paradigm, two-way ANOVA revealed significant group differences in the duration of freezing (F_7,126_ = 4.15, *p* < 0.0001, Figure 4D). Post hoc analysis revealed a significant decrease in freezing duration in both untreated and vehicle-treated groups (*p* = 0.042 and *p* = 0.023) in comparison with control untreated and vehicle-treated animals. Citalopram and celecoxib counteracted this effect (*p* = 0.76 and *p* = 0.65), as compared with the stressed untreated and stressed vehicle-treated animals, respectively. Two-way ANOVA revealed significant group differences in the ratio of good to poor learners (F_7, 126_ = 2.39, *p* < 0.0001, Figure 4E). Two-tailed exact Fisher tests showed that the percentages of poor learners, defined as mice with freezing scores below 40%, were significantly lower in the citalopram-treated stressed group and celecoxib-treated stressed group than in the vehicle-treated group and untreated stressed group, respectively (*p* = 0.024 and *p* < 0.0001). In the citalopram-treated stressed group, 12 out of 25 mice were poor learners, 48%; in the celecoxib-treated stressed group, 5 out of 23 mice were poor learners, 22%; in the untreated stressed group, 16 out of 26 were poor learners, 62%; and in the vehicle-treated stressed group, 14 out of 20 mice were poor learners, 70%; Figure 4E). Post hoc analysis revealed a significant reduction in the number of poor learners in both citalopram-treated and celecoxib-treated groups (*p* = 0.028 and *p* = 0.009) in comparison with stressed untreated and stressed vehicle-treated animals.

### 2.4. Acute Administration of Celecoxib Reduces Floating in the Porsolt Test

**Figure 5:**
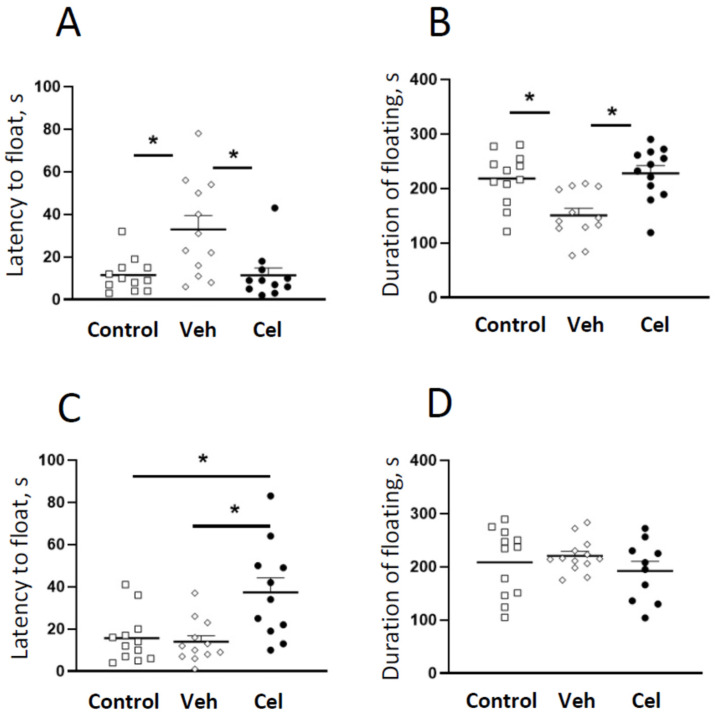
Effects of celecoxib treatments on behavior in the second session of Porsolt test. Celecoxib pre-treatment carried out 0.5 h before the first swimming session, prevented a decrease in latency to float (**A**) and the increase in duration of floating (**B**) in the second swimming session (* *p* < 0.05 vs. vehicle-treated group, one-way ANOVA and post hoc Tukey test). Celecoxib pre-treatment carried out 2 h after the first swimming session prevented the increase in latency before floating (**C**), and did not alter the duration of floating (**D**) in the second swimming session (* *p* < 0.05 vs. vehicle-treated group, one-way ANOVA and post hoc Tukey test).

One-way ANOVA revealed significant effects of celecoxib injected 30 min prior to the first session on the latency before floating (F_2,32_ = 7.509, *p* = 0.0021) and the duration of floating in the day 2 session (F_2,32_ = 9.46, *p* = 0.006 Figure 5A). This treatment significantly affected latency before floating (F_2,32_ = 7.835, *p* = 0.0017, Figure 5B). 

No significant difference was observed when the treatment was applied 120 min after the first session on the duration of floating in the second session (F_2,32_ = 0.84, *p* = 0.439). Celecoxib treatment given between the two sessions had no effect on the duration of floating (*p* = 0.406 vs. vehicle-treated group, Tukey post hoc test, Figure 5C) but increased the latency before floating (*p* = 0.032 vs. vehicle-treated group, Figure 5D).

## 3. Discussion

The present work revealed the over-expression of COX-2 and Iba-1 in the dentate gyrus and CA1 area and downregulation of Ki67 in the subgranular zone in the hippocampi of anhedonic (susceptible), but not non-anhedonic (resilient), mice, suggesting that these changes are may underpin the mechanisms of susceptibility to stress-induced anhedonia. We found a significant decrease in the percentage of anhedonic animals among celecoxib-treated stress mice, and a shortened duration of floating in celecoxib-treated animals in the Porsolt test, which further highlights a potential role for COX-2 in the mechanisms of depression and points to the therapeutic potential of its inhibition.

The results showed that susceptibility, but not resilience to stress-induced anhedonia, a core symptom of depression, is associated with an over-expression of COX-2 in neurons in the CA1 area and dentate gyrus, but not in the CA3 area, of the hippocampus in chronically stressed mice. The changes were also coincident with the increases in the numbers of Iba-1-positive cells in the hippocampus and a reduction of Ki67-positive cell number in the subgranular zone, suggesting increased microglial activation and suppressed cell proliferation in mice susceptible to a depressive-like syndrome. No such changes were evident in mice resilient to stress-induced anehdonia. We also found in the CMS study that chronic administration of selective COX-2 inhibitor celecoxib counteracted the development of the stress-induced depressive-like syndrome, lowered the percentage of anhedonic mice in the cohort, and normalized floating and hippocampal-dependent contextual learning behaviors in the stressed group of animals. The effects were similar in magnitude to those induced by citalopram administration. Bolus pre-treatment with celecoxib decreased floating behavior in the Porsolt test, further confirming the antidepressant effect of celecoxib. Together, these studies suggest a crucial role for hippocampal COX-2 activation in the mechanisms leading to susceptibility to a depressive-like syndrome and demonstrate the antidepressant activity of its inhibition with celecoxib, which is comparable to the widely used SSRI citalopram.

Our findings indicate a relationship between the COX-2 over-expression in the hippocampus and individual susceptibility to the depressive-like syndrome. Generally, it further supports the view that “neuroinflammation” contributes to an individual’s predisposition to MDD [64,75]. Our results are in keeping with previous studies of Song et al. [61] who, using the 5-week CMS and LPS challenge to model depression in Wistar rats, reported elevated production of COX-2 and PGE2 in dendritic spines [35,101], in the CA1 area and dentate gyrus of the hippocampus, which were associated with decreased dendritic plasticity, oxidative stress, and depressive-like behaviors [61]. The normalizing effects of antioxidant treatment with N-acetylcysteine on these outcomes, together with our earlier reports linking susceptibility to CMS-induced anhedonia with decreased brain activities of catalase and superoxide dismutase activity in mice [90], suggest that oxidative stress may mediate the effects of over-expressed COX-2 on anhedonia development. As such, the beneficial effects of celecoxib on depressive features and hippocampus-dependent memory in the fear conditioning paradigm are likely to be due to its normalizing effects on oxidative stress and cellular remodeling in the CA1 zone and dentate gyrus of hippocampal formation. Altered COX-2 expression in the hippocampus was shown to modulate its plasticity and LTD mechanisms, agreeing with earlier electrophysiological studies [102].

Previous studies with CMS variants stratifying mice for their susceptibility to stress-induced anhedonia showed that it can be associated with expression changes of several molecular and cellular markers of inflammation that are not displayed by resilience to anhedonic animals [22,95,103,104,105]. For example, CMS-exposed susceptible-to anhedonia-mice revealed significant elevations of COX-1 and IDO expression in the midbrain raphe region, suggesting a possible interaction of neuroinflammation with altered 5-HT transmission-relation mechanisms [95]. Anhedonic, but not resilient mice, showed an over-expression of TNF mRNA in the prefrontal cortex and an elevated number of Iba-1-positive cells in this brain structure [95]. These studies found similar increases in corticosterone blood levels, an important indicator of hyperactivity of the HPA axis in depressed patients [104,106] that in the context of the results reported here may be interpreted as a sign of dysregulation of COX-2 expression by glucocorticoids in a susceptible cohort of mice. Our data reporting the over-expression of inflammatory mediators in a susceptible depressive syndrome cohort of animals are in keeping with clinical data from depressed patients [107,108,109].

The functional effects of IL-1β in the CNS, which include sickness behavior, were also shown to be antagonized by treatment with a selective COX-2 inhibitor [110]. While the antidepressant effects of celecoxib were earlier shown in CMS mice and other depression models in rats, these experiments did not compare the effects of its pharmacological inhibition against the effects of standard antidepressants [59,61,63,111].

The current study revealed similar antidepressant-like activity of selective COX-2 inhibitor celecoxib and that of broadly used SSRI citalopram, suggesting that selective COX-2 inhibitors might be exploited to treat MDD. In comparison with the inhibition or genetic deletion of COX-1, which also counteracts the development of the depressive syndrome [112,113] and is functionally related to COX-2 [114,115], COX-2 inhibitors may display better compliance, since the constitutively expressed COX-1 is responsible for the maintenance of peripheral physiological functions and its inhibition causes significant side effects [110]. To date, several COX-2-selective inhibitors (coxibs) that have been used for the treatment of arthritis, post-operative pain, headaches, and inflammatory diseases of the brain and peripheral tissues have been developed [116]. However, due to their cardiovascular safety profiles, selective COX-2 inhibitors rofecoxib and valdecoxib were withdrawn from the market in 2005, whereas celecoxib is not reported to exhibit cardiovascular side effects, thereby remaining an FDA-approved drug. In any case, high affinity and selective coxibs can serve as promising prototypes in the development of novel, safe, and effective compounds that can be potentially beneficial for MDD patients [117].

Depressed patients display increased serum levels of pro-inflammatory cytokines, including TNF-α [108,118], that can trigger the activation of COX-2 [119,120], underlying the beneficial effects of treatment with celecoxib in previously reported clinical trials. Conversely, several studies have shown that antidepressants exert immunomodulatory properties suppressing low-level inflammation that may affect the human immune system and may partly contribute to their efficacy [121]. The inconsistences with clinical studies using celecoxib and the accumulating clinical evidence of heterogeneity among MDD patients in the manifestation of low-degree inflammation argue for the refinement of anti-inflammatory treatment strategies in depression. It has been suggested that inflammatory components may be used to characterize a specific subgroup of patients with MDD; e.g., high baseline levels of CRP have been linked to greater depressive symptom severity in general and specific symptoms, such as bad mood, little interest, little activity, suicidality, and poor cognitive performance [122]. PET markers of COX-2, which are currently under development, may also potentially be useful [38]. This approach may help to identify those subgroups of MDD patients who may benefit from a targeted, and thus more effective, treatment approach. Together, targeting inflammatory markers such as COX-2 would likely be a move towards more advanced personalized treatment of depression.

## 4. Materials and Methods

### 4.1. Animals 

Studies were performed using 3-month-old male C57BL/6N mice. Three-month-old male CD1 mice were used as intruders for social defeat stress and 2.5 month-old Wistar rats were used for predator stress. All animals were from Janvier, Charles River, France. C57BL/6J mice were housed individually for 10–14 days before the start of experiments; CD1 male 3-month-old mice were housed five per cage during the study; rats were housed in groups of five before the experiment and then individually. Animals were kept under a 12-h light–dark cycle (lights on: 19:00 h) with food and water ad libitum, using controllable laboratory conditions (22 ± 1 °C, 55% humidity). All experiments were carried out in accordance with the European Communities Council Directive for the care and use of laboratory animals 2010/63/EU upon approval by the Ethical Committee of C. Bernard University 08-2008-2011RC and MSMU #11-18-2018/2019 on animal care and welfare, and were compliant with ARRIVE guidelines (http://www.nc3rs.org.uk/arrive-guidelines, 2 January 2022). 

### 4.2. Chronic Stress Experiments 

This study used a previously validated 4-week stress protocol [90] that was adapted from previously described method [88,93]. The stress regimen comprised of a nighttime rat exposure and the daytime application of three stressors—a social defeat, restraint stress, and tail suspension, a combination of which was applied in a semi-random manner (for details see Supplementary Material). Briefly, between the hours of 09:00 and 18:00, three stressors per day were employed in the following sequence: social defeat for 30 min, restraint stress for 2 h, and tail suspension for 40 min with an inter-session interval of at least 4 h.

With the drug-free stress protocol, 12 naive control mice were used, and 20 mice were subjected to stress. At the baseline, control and stress groups of mice were balanced upon their sucrose preference, body weight, and social behavior (non-aggressive or aggressive) as described elsewhere ([88,90,123,124]; see also below). The sucrose preference test was repeated on the 2nd and 4th weeks of stress exposure. After the termination of the stress procedure, the latter group of mice was assigned to resilient and anhedonic cohorts according to their sucrose preference and studied in the forced swim test (see below). Sucrose preference two-bottle test was performed during dark phase of animals’ cycle, between 09.00–17.00, as described elsewhere [97]. All mice were tested behaviorally one day after the termination of chronic stress, i.e., starting 24 h after the last rat exposure stress session and sacrificed 36 h after the termination of stress (Figure 1A; see below). The sacrificed subgroups of control, resilient, and anhedonic animals were used to study the hippocampal COX-2 gene expression (each group was comprised of 7 mice) or immunohistochemical staining of COX-2-positive cells (5 controls, 5 resilient, and 5 anhedonic mice were used); remaining animals were used in the pilot studies.

In a follow-on chronic stress study, 58 mice were assigned to a non-stressed control group. Among them, 13 mice constituted each control group that was not treated or received i.p. injection of DMSO-vehicle; 16 control mice per group were treated with daily i.p. injections of citalopram (15 mg/kg/day) or celecoxib (30 mg/kg/day). Among the animals subjected to stress, 26 of them were untreated, 22 received vehicle, 32 were treated with citalopram (15 mg/kg/day), and 25 had daily injections of celecoxib (30 mg/kg/day) during the 7 days prior the onset of stress and during entire stress procedure, as described elsewhere ([93]; see Supplementary data). Mice were assigned to these groups after baseline measurements and subjected to the stress procedure and behavioral tests, as in the preceding CMS study (Figure 1B; see below). The percentage of animals that were categorized as susceptible to stress-induced anhedonia was calculated. In addition, mice were studied for their hippocampus-dependent memory in the fear conditioning paradigm of contextual learning, as described elsewhere [98,125]. All groups of mice were trained on the second day of a post-stress period in the fear conditioning chamber and tested for a recall approximately 24 h thereafter.

### 4.3. A Study with the Porsolt Test

Mice were subjected to two swimming sessions with an interval of 24 h, where the i.p. administration of vehicle or celecoxib (30 mg/kg/day) was carried out 30 min prior the first swim session (Figure 1C) or 2 h thereafter (Figure 1D; see below). Twelve control untreated and 12 vehicle-treated mice were used in each study; celecoxib-treated mice constituted 11 mice in each experiment.

### 4.4. Chronic Stress Procedure and Determination of Anhedonia 

In this study, the chronic stress procedure was applied as described previously [98]. Shortly, the mice were subjected to 4 different stressors (rat exposure, restraint stress, social defeat, and tail suspension procedure) over 4 weeks as described elsewhere ([90,95,126]; see Supplementary File). To assess the hedonic state in mice, the sucrose preference test was performed one week before the experiment (baseline measurement), on the 2nd week of stress and 4 weeks after the beginning of the stress procedure, (see below). Stressed mice that after the 4th week of stress showed a decrease of sucrose preference below 65%, were assigned to the anhedonic group, accordingly to the previously proposed criterion of anhedonia [88]. The remaining animals were considered as non-anhedonic (resilient to stress-induced anhedonia). Applied criterion of anhedonia was based on our previous results, which demonstrated that mice with a sucrose preference ≤65% showed a depressive-like syndrome, consisting in increased floating and decreased exploration, whereas stressed mice with a sucrose preference above this value did not display this behavioral phenotype [22,92,127]. 

### 4.5. Sucrose Preference Test

Mice were given eight hours of free choice between two bottles of either 1% sucrose or standard drinking water. At the beginning and end of the period, the bottles were weighed and consumption was calculated. The beginning of the test started with the onset of the dark (active) phase of animals’ cycle, i.e., at 9.00). To prevent the possible effects of side-preference in drinking behavior, the position of the bottles in the cage was switched at 4 h, halfway through testing. No previous food or water deprivation was applied before the test. To minimize the spillage of liquids during sucrose test, bottles were filled in advance and kept in the up-side-down position for at least 12 h prior to testing. In order to balance the air temperature between the room and the drinking bottles, they were kept in the same room where the testing takes place. This measure prevents the physical effect of liquid leakage resulting from growing air temperature and pressure inside the bottles, when they are filled with liquids which are cooler than the room air. Preliminary tests showed that with this method the error of measurement does not exceed 0.1 mL. In order to decrease variability in sucrose consumption during the very first sucrose test (baseline measurement), a day before, animals were allowed to drink 2.5% sucrose solution in a one-bottle paradigm for 2 h.

Percentage preference for sucrose is calculated using the following formula: *Sucrose Preference = Volume (Sucrose solution)/(Volume (Sucrose solution) + Volume (Water))* *× 100*. No mice from control groups ever exhibited a preference for sucrose of <65% and, accordingly, mice exhibiting a sucrose preference of <65% were defined as susceptible. Mice that had undergone stress but maintained a sucrose preference of >65% were defined as resilient. Other conditions of the test were applied as described elsewhere [22,89,92].

### 4.6. Forced Swim Test

Two days after the termination of stress procedure, mice were tested in the forced swim test. Mice were introduced to a transparent pool (20 cm × 35 cm × 15 cm) filled with warm water (30 °C, height 9.5 cm) lit by red light for 2 min. The duration of floating behavior, defined as absence of directed movements of animals’ heads and bodies, was estimated as described elsewhere [123,128]. 

### 4.7. Fear Conditioning Paradigm

The apparatus (Technosmart, Rome, Italy) consisted of a transparent plastic cubicle (25 × 25 × 50 cm) with a stainless-steel grid floor (33 rods, 2 mm in diameter). A single alternating electric current (AC, 50 Hz; 0.7 mA, 1 s, Evolocus LLC, Tarrytown, NY, USA) was delivered after a 2-min acclimatization period. After delivery of the current, the mouse was immediately placed back in the home cage. Freezing behavior was scored by visual observation during a test of memory recall that was carried out 24 h later as described elsewhere [96,98]. The occurrence of freezing behavior was assessed every 10 s for 180 s; each 10-s score was assigned to a freezing or non-freezing period, and the percentage of time spent in freezing was calculated. Mice spent in freezing ≥40% of time were defined as “good learners” as described elsewhere [129].

### 4.8. A Two-Day Forced Swimming Porsolt Test and Drug Administration

All sessions were 6-min long and were performed by placing a mouse in a transparent cylinder (Ø 17 cm) filled with water (23 °C, water height 13 cm, height of cylinder 20 cm). On day 2, the duration of floating behavior that was defined by the absence of any directed movements of the animals’ heads and bodies, was scored manually using criteria, which were previously validated by automated scoring with Noldus EthoVision XT 8.5 (Noldus Information Technology, Wageningen, The Netherlands) and CleverSys (CleverSys, Reston, VA, USA) as described elsewhere [100,130]. The latency before floating and time spent floating were recorded.

### 4.9. Administration of Drugs

Citalopram (Cipramil: Lundbeck, Copenhagen, Denmark) was dissolved in sterile water for injection. Celecoxib (Celebrex: Pfizer, St. Louis, MO, USA) was dissolved in a vehicle containing 34% Hydroxypropyl-ß-cyclodextrin (Sigma, Steinheim am Albuch, Germany) and 10% DMSO (Sigma, Steinheim am Albuch, Germany). Mice were intraperitoneally injected with either DMSO-vehicle, citalopram, or celecoxib. The dose of citalopram was based on previous studies showing the efficacy under employed settings [93,123]. The dose of celecoxib was defined by previous reports [59,131].

### 4.10. Culling and Brain Dissection

Mice were terminally anaesthetized with isoflurane inhalation and sacrificed by cervical dislocation for a subsequent material collection. For gene expression assay, mice were perfused with ice-cold saline via left ventricle, brains were removed, hippocampi were dissected and stored at −80 °C until use as described elsewhere [132]. For imunohistochemical study, mice were perfused with 10 mL ice-cold saline followed by 4% paraformaldehyde via left ventricle, brains were removed, post-fixed in PFA for 12 h and cryoprotected in 30% sucrose for 12 h and then embedded in a mold filled with OCT compound and snap-frozen in dry ice-cooled isopentane. Samples were stored at −30 °C until used as described elsewhere [133,134].

### 4.11. RNA Extraction and RT-PCR

First strand cDNA synthesis was performed using random primers and Superscript III transcriptase (Invitrogen, Darmstadt, Germany); 1 μg total RNA was converted into cDNA. Quantitative PCR for COX-2 gene and the housekeeping gene glyceraldehyde 3-phosphate dehydrogenase (GAPDH) was performed using the SYBR Green master mix (Bio-Rad Laboratories, Philadelphia, PA, USA) and the CFX96 Real-time System (Bio-Rad Laboratories, Philadelphia, PA, USA). Sequences of primers used are: COX-2 (5′-CCGTGCTGCTCTGTCTTAAC-3′ and 5′-TTGGGAACCCTTCTTTGTTC-3′), GAPDH (5′-CTGCACCACCAACTGCTTAG-3′ and 5′-GGGCCATCCACAGTCTTC-3′). Data were normalized to GAPDH mRNA expression and calculated as relative-fold changes compared to control mice as described elsewhere [100,134]. Results of RT-PCR measurement were expressed as Ct values, where Ct is defined as the threshold cycle of PCR at which amplified product was 0.05% of normalized maximal signal. We used the comparative Ct method and computed the difference between the expression of the gene of interest and GAPDH in each cDNA sample (2^−ΔΔCt^ method). Data are given as expression-folds compared to the mean expression values in control mice.

### 4.12. Immunohistochemical Analysis of COX-2-Positive Cells in the Brain

Immunostaining with COX-2, NeuN, Iba-1, and Ki67 antibodies and image analysis in the hippocampus were performed as described elsewhere [135]. Coronal 10 μm-thick sections were cut on a cryostat microtome (Leica Biosystems, Nussloch, Germany) and mounted on gelatin-coated slides. Hippocampal sections were taken from lateral 3.6 to lateral 0.4 mm along the medial lateral axis up to bregma (Paxinos and Franklin, 2001). Slides were washed in PBS and blocked for non-specific protein binding with 10% goat serum in PBS for 1 h. Then, sections were incubated with primary antibody (COX-2: 1:1000, ab178846, Abcam, Cambridge, UK; NeuN: 1:1000, MAB377, Millipore, Billlerica, MA, USA; Iba1: 1:800, ab5076, Abcam, Cambridge, UK; Ki67: 1:500, ab15580, Abcam, Cambridge, UK) in 1% normal goat serum at 4 °C for 12 h. Visualization was performed using secondary antibodies: anti-rat-Alexa Fluor 594 (1:500, Abcam, Cambridge, MA, USA), anti-rabbit-Alexa Fluor 488 (1:500, Abcam, Cambridge, MA, USA), and anti-chicken-Alexa Fluor 647 (1:500, ThermoFisher, Abingdon, UK) in 1% serum in PBS (Vector Laboratories, Burlingame, CA, USA) for two hours at room temperature. To visualize the nuclei of the hippocampal cells, sections were co-stained with 4′,6-diamidino-2-phenylindole (DAPI) (Santa Cruz Biotechnology, Santa Cruz, CA, USA). Immunostaining was examined using a light microscope Leitz Dialux 20 (Leica, Wetzlar, Germany) and digital camera Basler ACE (Basler Group, Ahrensburg, Germany). The areas of CA1 and CA3 zones, hilus, and the subgranular zone were specifically delineated according to the Paxinos atlas. Cell counting was carried out using ImageJ software. Three sections per each structure per animal were analyzed. 

### 4.13. Statistical Analysis

Data were analyzed with a statistical software package (Statistica 10.01, Chicago, IL, USA). ANOVA test followed by post hoc Tukey test was used for data analysis. One-way and two-way ANOVA were applied where appropriate. Assuming equal variability of differences no Geisser-Greenhouse correction was applied. Qualitative data were analyzed by the two-tailed Fisher’s exact test. The level of confidence was set to 95% (*p* < 0.05). 

## 5. Conclusions

Our findings demonstrate that the up-regulation of COX-2 expression in the CA1 zone and dentate gyrus of the hippocampus is associated with individual susceptibility to stress-induced depressive syndrome. We also report similar efficacy of antidepressant action of the selective inhibitor of COX-2 celecoxib compared to the SSRI citalopram in the CMS mouse model. In light of the considerable side effects reported for SSRIs and other classic antidepressants, resulting in premature discontinuation of the medication in over 70% of individuals [136], the use of COX-2 inhibitors would likely be beneficial. This add-on therapy might become particularly valuable as soon as appropriate clinical guidance for the use of anti-inflammatory therapy and new potentially safe COX-2 inhibitors will be developed. 

## Data Availability

Data are available upon request.

## Supplementary Materials

The following supporting information can be downloaded at: https://www.mdpi.com/article/10.3390/ijms23042061/s1.
