# Supplementary material for: Hippocampal Over-Expression of Cyclooxygenase-2 (COX-2) Is Associated with Susceptibility to Stress-Induced Anhedonia in Mice
Source: Int J Mol Sci. 2022 Feb 13;23(4):2061. doi: 10.3390/ijms23042061 (PMC8879061; doi:10.3390/ijms23042061)
Supplement: Supplementary file 1 [file ijms-23-02061-s001.zip › ijms-1555573-supplementary.pdf]

Supplementary File

Chronic stress procedure

Rat exposure while in a small container

Mice were introduced into cylindrical containers (Open Science, Moscow, Russia), which were placed into a rat home cage during 15h (over-night, from 18h00 to 9h00). Containers were made from customized transparent plastic, size 15cm x Ø 8 cm, with holes in covers (Ø < 0.5cm), which ensured protection of the mouse from the rat, but allowed visual and odor contact. During the weekends, mice were kept in their home cage, which were situated on top of the rat cages.

Restraint stress

Animals were placed inside a plastic tube (internal diameter 26 mm) for 2 h during the dark phase of the light cycle and kept in a dark experimental room.

Tail suspension stress

Mice were submitted to the tail suspension procedure by hanging them by their tails in a tail suspension system (Bioseb, France) for about 6 min daily. The procedure was done during the dark phase of the animals' light cycle.

Social defeat stress

Social defeat procedures took place during the dark phase; to enable a visual control over the resident-intruder confrontation, the test was carried out under red light. In a preliminary test, aggressive individuals of the CD1 mouse strain that were able to attack the counter-partners in less than 60 sec without injuring them were selected for this procedure; these animals were introduced in the home cages of mice from the stress group during social defeat sessions for 5 min. During social defeat stress, test mice typically showed flight response, submissive posture and vocalization. Pairs of animals were carefully observed in order to exclude any physical harm. In rare cases of its incidence, aggressive individuals were immediately removed from the cage of resident mice. After a 5-min period of social defeat C57BL/6 mice were introduced into small containers and again inside the CD1 cage, where they stayed for a 3 h-period. Thereafter, a 5-min social defeat procedure was repeated again. In order to randomize the procedure, the same pairs of C57Bl6 and CD1 mice were never put together.
